# The levels of miR-155 in epilepsy patients: a meta-analysis

**DOI:** 10.3389/fneur.2025.1630581

**Published:** 2025-08-13

**Authors:** Yi Wang, Cong Chen, Chengyu Ya, Jiangwei Chen, Bingfang Lu, Jinwen Liu, Qiong Wu, Limei Diao, Huihua Liu

**Affiliations:** ^1^Graduate School of First Clinical Medicine College, Guangxi University of Chinese Medicine, Nanning, Guangxi, China; ^2^Department of Neurology, Putian Hanjiang Hospital, Putian, Fujian, China; ^3^Department of Pediatric Rehabilitation, Xinyang Central Hospital, Xinyang, Henan, China; ^4^Department of Neurology, Guangxi Zhuang Autonomous Region Brain Hospital, Liuzhou, Guangxi, China

**Keywords:** epilepsy, miR-155, neuroinflammation, biomarker, meta-analysis

## Abstract

**Background:**

Neuroinflammation plays an important role in the development and progression of epilepsy. It can be both a result and a potential cause of those seizures. MiR-155 plays a crucial role in inflammatory responses, and its expression is upregulated under various neuroinflammatory conditions. This study aims to compare the expression levels of miR-155 in the brain tissue or serum of epilepsy patients and healthy controls, assessing the correlation between miR-155 levels and epilepsy.

**Objective:**

The meta-analysis evaluates to assess and compare the levels of miR-155 in the brain tissue and serum of epilepsy patients and healthy controls.

**Methods:**

The databases PubMed, Embase, Cochrane Library, CNKI, VIP, and WanFang DATA were searched from inception until Dec 30, 2024 by two researchers. The relative expression level of miR-155 in the tissues or serum was the primary outcome of the search. After extracting data independently, the two researchers used the Cochrane Collaboration’s tool for assessing risk of bias. All data were analyzed using Review Manager (V.5.4.1) statistical software.

**Results:**

This meta-analysis (nine studies, 394 patients) reveals elevated miR-155 in epilepsy patients vs. controls (SMD = 1.62, *p =* 0.001), especially in brain tissue. Subgroups confirm consistency across ages/regions. Subgroup analysis revealed no significant differences by country (*p =* 0.31), age category (*p =* 0.63) and sample source (*p =* 0.15), indicating robust consistency of the primary findings. MiR-155 may serve as a neuroinflammatory biomarker in epilepsy.

**Conclusion:**

Compared to healthy controls, the relative expression level of miR-155 in the brain tissue or serum of epilepsy patients increased.

**Systematic review registration:**

https://www.crd.york.ac.uk/prospero/, identifier: CRD42024558255.

## 1 Introduction

Epilepsy is a chronic, non-communicable brain disorder that affects individuals of all ages and is characterized by recurrent seizures resulting from abnormal electrical signals produced by damaged brain cells (1). Approximately 70 million people worldwide have epilepsy, with 80% living in low and middle-income countries. It is estimated that 5 million people are diagnosed with epilepsy each year, with a prevalence rate of 6–7%. In China, there are about 9 million epilepsy patients, with around 5–6 million experiencing active epilepsy (2). Poverty, lack of medical resources, and health facilities are major factors affecting the effective treatment of epilepsy patients in above-mentioned countries (3). As a chronic neurological disorder, epilepsy remains a significant public health issue affecting millions of people globally. Patients with epilepsy require long-term medical care and management, but due to the uneven distribution of healthcare resources, many of them do not have access to timely and effective treatment, leading to exacerbation of their condition and further aggravating the pressure on the public health system.

Currently, the diagnosis of epilepsy primarily relies on clinical assessment, including medical history, seizure description, and electroencephalogram (EEG) (4). Imaging techniques, such as magnetic resonance imaging (MRI) and computed tomography (CT), also play a crucial role in identifying potential brain structural abnormalities associated with epilepsy (5, 6). However, despite the availability of these tools, there are still considerable challenges in accurately diagnosing epilepsy, especially when traditional test results are inconclusive. This is where biomarkers emerge as promising adjuncts in the diagnosis and treatment of epilepsy. Several biomarkers such as neurotransmitters and inflammatory markers have been identified for use in diagnosis or monitoring of epilepsy.

MicroRNAs (miRNAs) are single-stranded RNAs universally present in eukaryotic organisms and serve as major regulators of gene expression (7). Individual miRNA can affect multiple proteins across different molecular pathways and networks. Among them, the study of miR-155 as a diagnostic marker for epilepsy gradually develops, with some evidence suggesting its potential in the diagnosis of epilepsy. First, miR-155 plays an important role in various inflammatory responses, and the inflammatory mechanism is believed to play a key role in the pathology of epilepsy (8, 9). Second, epilepsy may not have obvious clinical manifestations in its early stages, especially during the interictal period, where routine tests may fail to capture abnormalities. However, abnormal expression of miR-155 may appear early or even before seizures occur, suggesting its potential to aid in early diagnosis. Nonetheless, there is still no direct evidence linking miR-155 to epilepsy. This meta-analysis aims to observe the expression levels of miR-155 in the brain tissue or serum of epilepsy patients compared to healthy controls, assessing the correlation between miR-155 levels and epilepsy.

## 2 Methods

This study adheres to the Preferred Reporting Items for Systematic reviews and Meta-Analyses (PRISMA) statement 2020 and has been registered on the PROSPERO website (registration number: CRD42024558255).

### 2.1 Search strategy and selection criteria

We researchers independently conducted a literature search in the PubMed, Embase, Cochrane Library, CNKI, WanFang DATA, and VIP databases for articles published prior to December 30, 2024. The search terms used were: (“epilepsy” OR “epilepsies” OR “seizure disorder” OR “seizure disorders”) AND (“miRNAc-155” OR “miR-155”). Inclusion criteria consisted of case–control that reported on the levels of miR-155 in the serum and tissue of epilepsy patients and control groups. Exclusion criteria included studies with overlapping populations, animal studies, case reports, systematic reviews, commentaries, letters, and reviews.

### 2.2 Data extraction

Two researchers (YW and LMD) independently extracted data from the included studies and cross-validated their results. They initially screened the literature by reviewing titles and abstracts for relevance, followed by a full-text review. Information extracted included the title, authors, publication year, country, article type, study population, research methodology, conclusions, sample size, age and gender of both epilepsy patients and controls, type of epilepsy, source of samples, and relative expression levels of miR-155. In cases of disagreement between the two reviewers, a third reviewer was consulted for adjudication. The literature was managed using EndNote (version X9; Thomson Reuters LLC, Philadelphia, PA, USA), and a manual review was conducted to finalize the selection of studies included in the review and meta-analysis. The risk of bias in randomized controlled trials (RCTs) was assessed using the Cochrane Risk of Bias tool (RoB 2), while the quality of non-randomized studies was evaluated using the Newcastle-Ottawa Scale (NOS).

### 2.3 Meta-analysis

We conducted the meta-analysis using Review Manager version 5.4. Given that the measurement methods across studies varied slightly, we calculated the effect size using the standardized mean difference (SMD). The SMD represents the difference in miR-155 levels between epilepsy patients and their corresponding controls. For studies with small sample sizes, we utilized Hedges’ g as the effect size measure to mitigate bias associated with small sample sizes. The I^2^ statistic was employed to describe the heterogeneity among the included studies; *p* < 0.00001 and an I^2^ value of 69% indicated significant heterogeneity among the studies. In light of this heterogeneity, we performed a random-effects analysis. Potential sources of heterogeneity included patient characteristics (sex, age, socioeconomic status) and study variations (geographical location, sample sizes). To explore the sources of heterogeneity, we conducted subgroup analyses.

## 3 Results

### 3.1 Study selection

**Figure 1:**
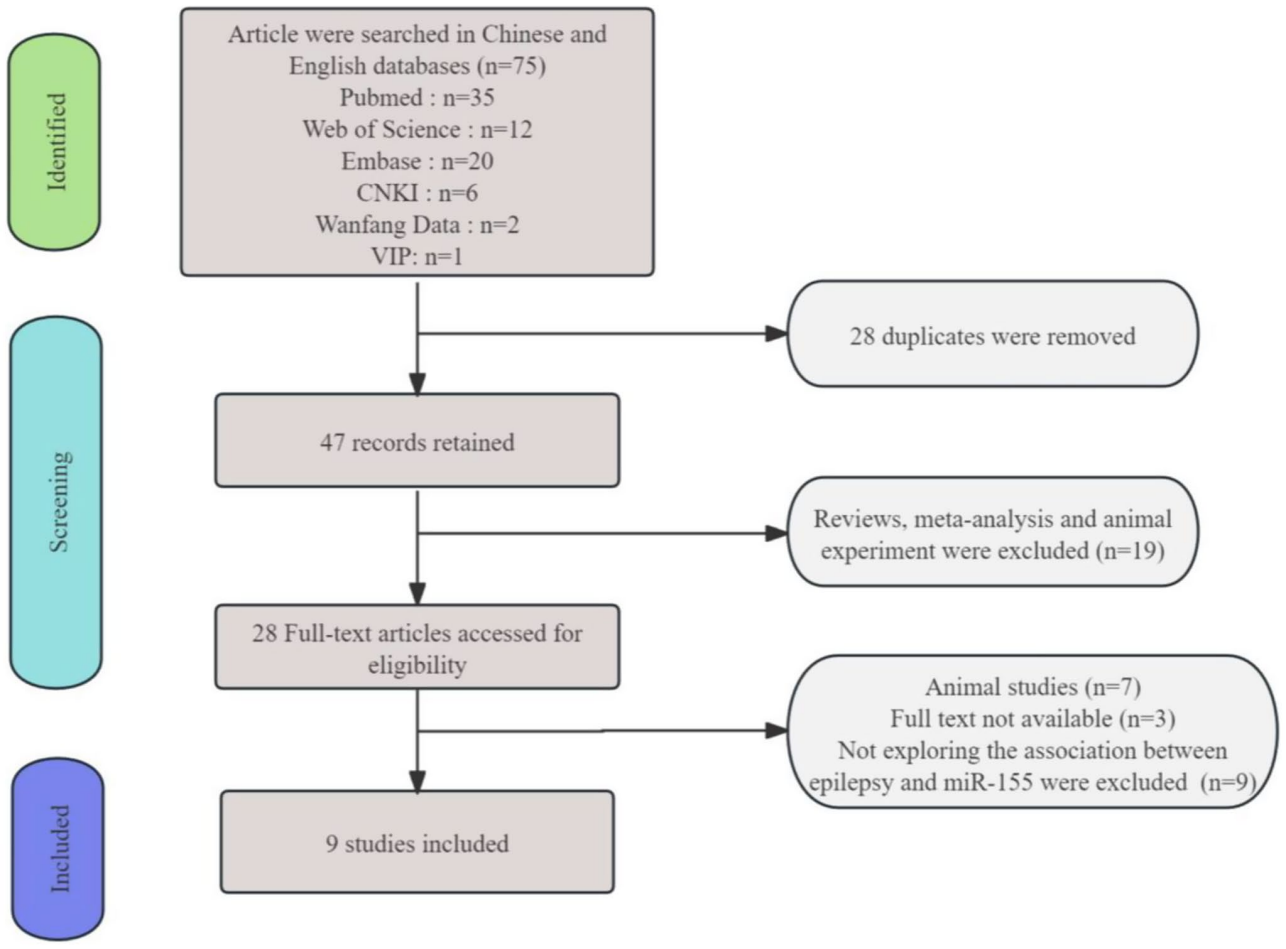
The diagram illustrating the systematic literature search.

By conducting a search in PubMed, Embase, Cochrane Library, CNKI, WanFang DATA, and VIP databases, we identified a total of 75 references. After excluding 28 duplicate articles, a detailed screening was performed on the remaining 47 publications. Among these, nine review articles, 1 meta-analysis and 9 articles were excluded due to missing data. Additionally, nine articles were removed because their results were not relevant to this analysis, and three articles had unavailable full texts with no response received after contacting the authors. In addition, we also screened seven animal experimental studies related to this analysis (10–16). Ultimately, a total of 9 studies were included in the analysis (17–25). The detailed process of study selection is illustrated in Figure 1.

### 3.2 Details of studies

The detailed information of the included studies is presented in Table 1. All included clinical trial studies were case–control studies, and no cohort studies were included as this research focuses on retrospective analysis. While the majority of studies originated from China (17–23) (*n* = 5), our analysis also included one Netherlands (24) study one South Korea study (25). The samples used for miR-155 measurement primarily consisted of serum and brain tissue. Among the nine included studies, two did not specify the age of the samples, three involved children epilepsy patients, and the remaining four targeted adults. The quality of the included studies ranged from 5 to 7, with a mean quality score of 5.89 (Table 2). All studies met the predefined inclusion criteria.

**Table 1:** Characteristics of the included studies.

Country	Sample sizes (TLE: CO)	Age category	Epilepsy type	Type of miRNAs	Sample source	Assay type	Mean age	miR-155 relative expression (Mean ± sd)	*p*-value
China	TLE: CO = 12:11	NA	TLE	miR-155	brain tissue	RT-qPCR	TLE (age, 9–69)	TLE:1.62 ± 1.18 CO:0.53 ± 0.66	<0.05
China	TLE: CO = 68:42	NA	TLE	miR-155	brain tissue	RT-qPCR	TLE (age, 9–69)	TLE:2.08 ± 1.81 CO:1.14 ± 0.52	<0.01
China	TLE: CO = 7:8	Adult	TLE	miR-155	brain tissue	RT-qPCR	TLE (age, 19–43) CO (age, 16–48)	TLE:5.6 ± 1.2 CO:3 ± 0.55	<0.01
Netherlands	TLE: CO = 16:6	Adult	TLE	miR-155	brain tissue	RT-qPCR	TLE (age, 24–49) CO (age, 25–86)	TLE:3 ± 1.28 CO:1 ± 0.42	<0.001
China	TLE: CO = 8:8	Minor	MTLE	miR-155	brain tissue	RT-qPCR	TLE (age, 8–13) CO (age, 8–12)	TLE:0.8 ± 0.17 CO:0.4 ± 0.1	<0.05
China	TLE: CO = 41:39	Adult	TLE	miR-155	brain tissue	RT-qPCR	TLE (age, 33–39) CO (age, 33–39)	TLE:1.1 ± 0.83 CO:0.22 ± 0.13	<0.05
China	TLE: CO = 23:8	Adult	TLE	miR-155	brain tissue	RT-qPCR	TLE (age, 15–80) CO (age, 15–80)	TLE:2.95 ± 1.05 CO:1.1 ± 0.08	<0.001
China	TLE: CO = 43:43	Minor	TLE	miR-155	Serum	RT-qPCR	TLE (age, 6–11) CO (age, 6–11)	TLE:1.82 ± 0.39 CO:1.18 ± 0.39	<0.05
Korea	TLE: CO = 8:3	Minor	TLE	miR-155	brain tissue	RT-qPCR	TLE (age, 7) CO (age, 6)	TLE:9.8 ± 2.69 CO:1.3 ± 0.64	<0.05

NA, Not applicable.

**Table 2:** The quality of the selected studies was assessed using the NOS.

Year	Studies	Selection	Comparability	Exposure	Total
2013	Ashhab, Muhammad	4	1	1	6
2014	Lee, Ji Yeoun	4	1	1	6
2017	L-G. Huang	4	0	1	5
2018	Li, Tao-Ran	4	1	1	6
2018	Zhang, Zhijie	4	1	2	7
2018	Korotkov, Anatoly	4	1	1	6
2019	Fu, Huajun	4	0	1	5
2021	Zhu, Zhujun	4	1	1	6
2022	Liu, Ya	4	1	1	6

### 3.3 Mean result between miR-155 and epilepsy

**Figure 2:**
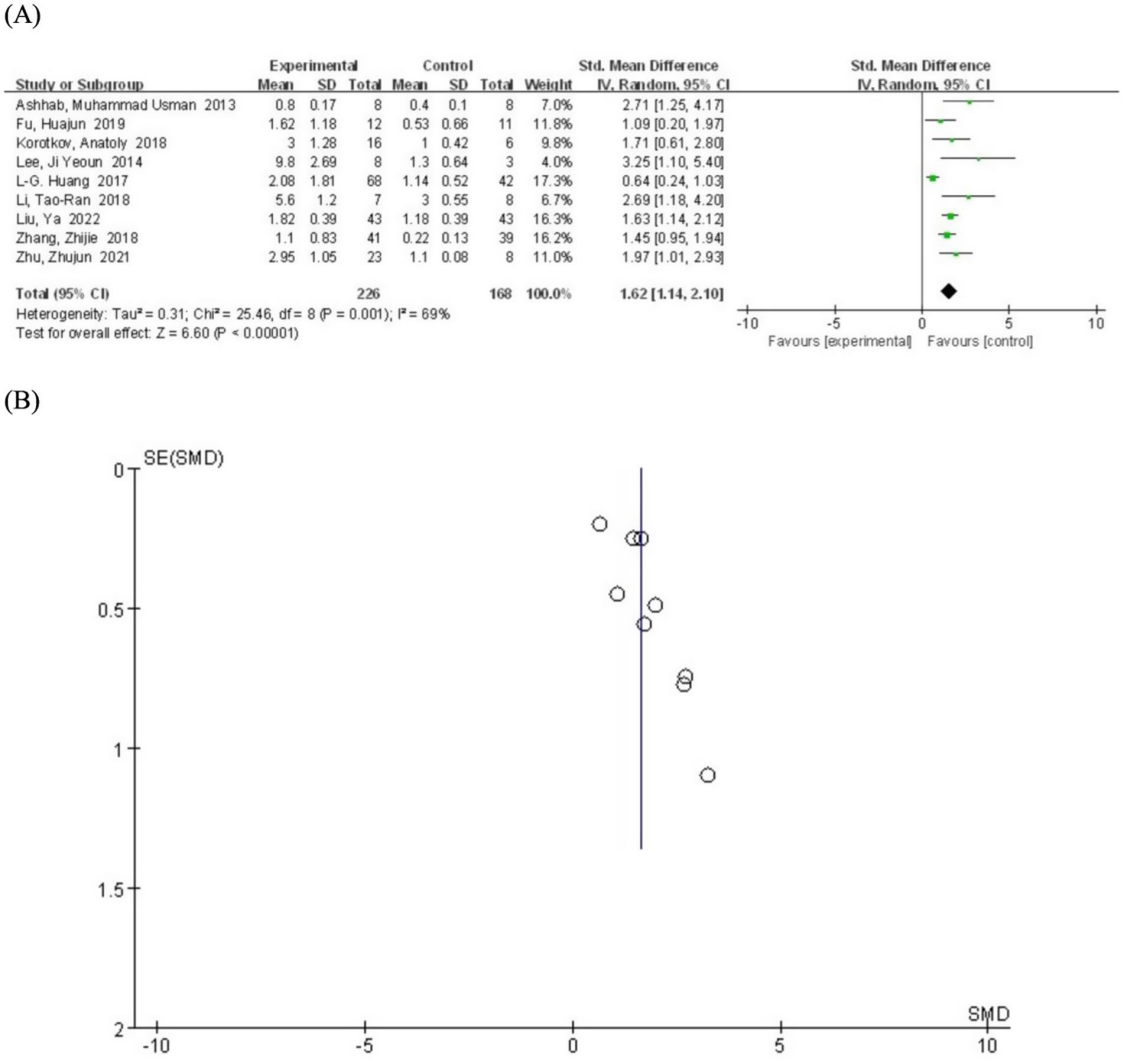
**(A)** Forest plot depicting the relationship between miR-155 level and epilepsy. **(B)** A funnel plot to assess publication bias.

We systematically generated forest plots encompassing data from 9 clinical trial studies, followed by comprehensive subgroup analyses. The meta-analysis revealed a statistically significant positive correlation between miR-155 relative expression levels and epilepsy (SMD = 1.62; 95% CI, 1.14–2.10, *p =* 0.001) (Figure 2). This finding is consistent with multiple lines of evidence demonstrating miR-155 involvement in cellular proliferation and apoptosis pathways, particularly in predicting the risk of post-traumatic epilepsy, thereby suggesting its crucial role in epileptogenesis (26). Notably, investigations focusing on children epilepsy populations have further substantiated miR-155’s regulatory function in epileptogenic processes (27). Moreover, emerging evidence from molecular biomarker correlation studies has identified miR-155 as a promising candidate biomarker for both acute seizure events and status epilepticus (28).

### 3.4 Subgroup analysis by country

**Figure 3:**
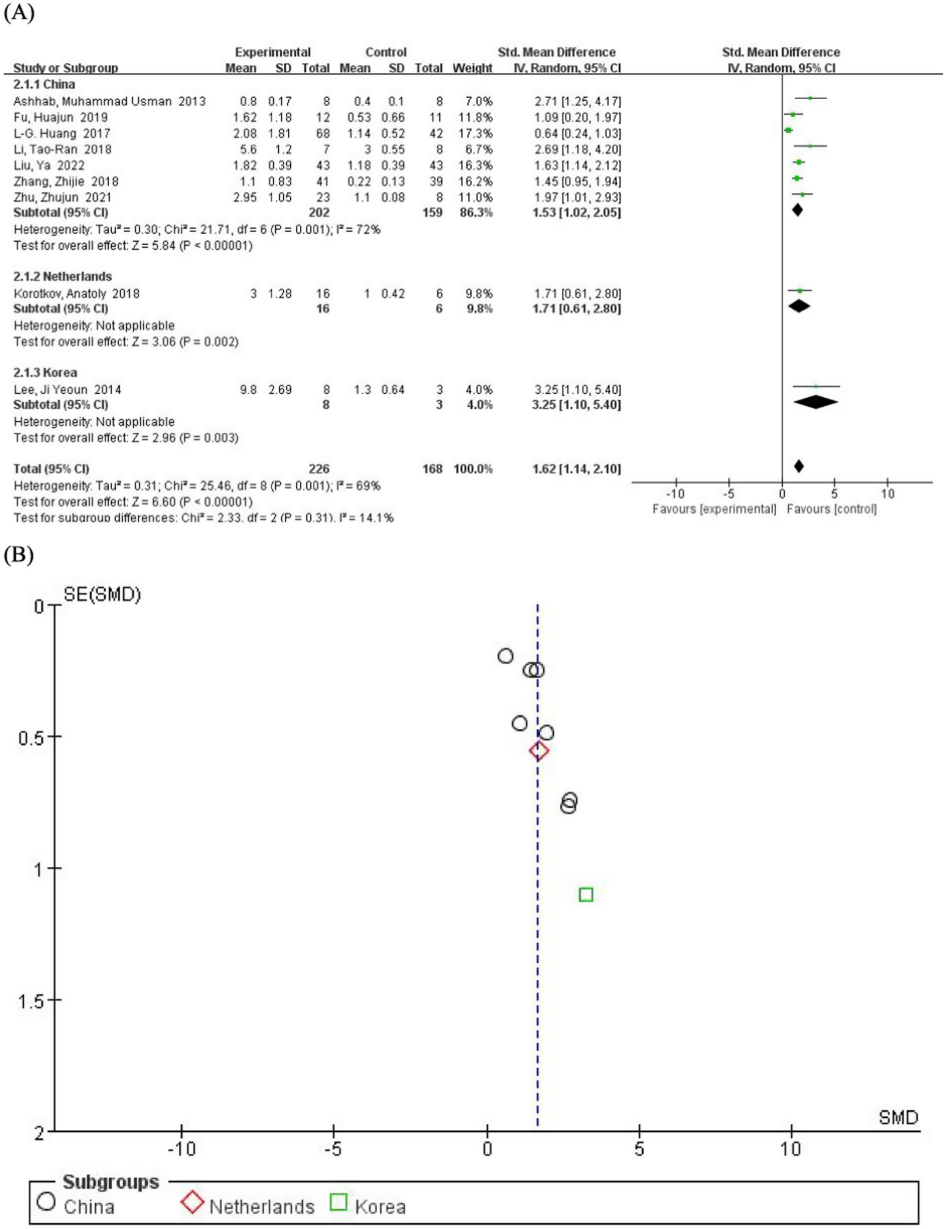
**(A)** Subgroup analysis by country demonstrating the association between serum miR-155 level and epilepsy. **(B)** A funnel plot to assess publication bias.

We conducted subgroup analyses based on the countries of the study participants to explore potential regional differences in intervention effects. The main results by country are as follows: the miR-155 levels in epilepsy patients were significantly higher than those in the control population in China (SMD = 1.53; 95% CI, 1.02–2.05; *p =* 0.001) (Figure 3), Netherlands (SMD = 1.71; 95% CI, 0.61–2.80; *p =* 0.002), and South Korea (SMD = 3.25; 95% CI, 1.10–5.40, *p =* 0.003). Although the data for the Netherlands and South Korea were derived from single studies (precluding calculation of I^2^ heterogeneity values), the pooled effect size from subgroup analysis (SMD = 1.62; 95% CI, 1.14–2.10; *p =* 0.001) demonstrates consistent elevation of miR-155 levels in epilepsy patients. Nevertheless, Future studies with larger sample sizes from these countries are needed to confirm this observation.

### 3.5 Subgroup analysis by age category

**Figure 4:**
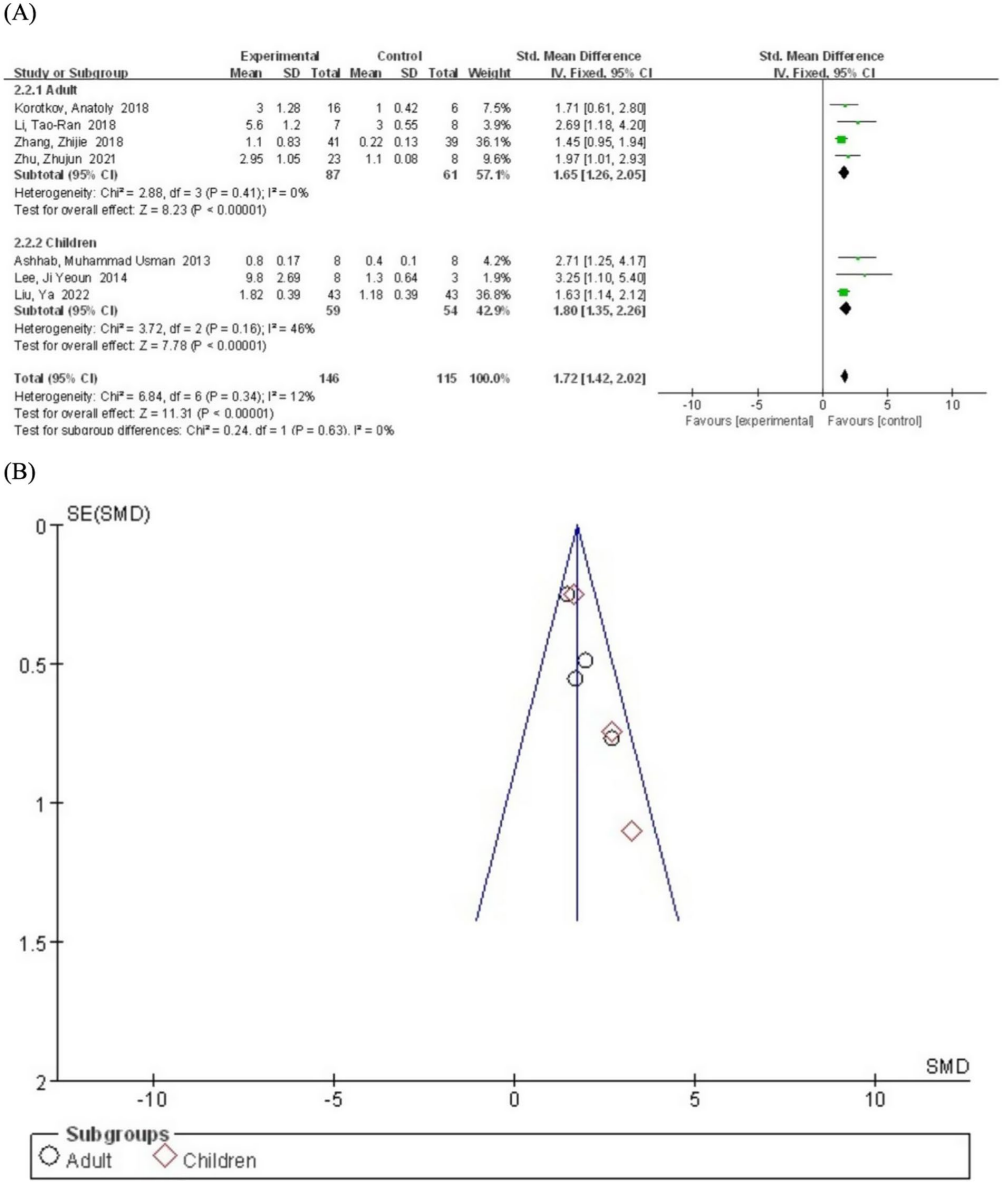
**(A)** Subgroup analysis by age category demonstrating the association between serum miR-155 level and epilepsy. **(B)** A funnel plot to assess publication bias.

We performed subgroup analyses based on age to evaluate the potential influence of age on miR-155 levels in epilepsy patients. The participants were divided into adults (≥18 years) and childrens (<18 years). The pooled analysis included four studies involving adult patients. The results demonstrated that miR-155 levels were significantly elevated in adult epilepsy patients compared to the control group (SMD = 1.65; 95% CI, 1.26–2.05; *p* < 0.00001) (Figure 4). The heterogeneity was negligible (I^2^ = 0%), indicating low variability. Three other included studies involving children patients. Similarly, miR-155 levels were significantly higher in children with epilepsy compared to the control group (SMD = 1.80; 95% CI, 1.35–2.26; *p* < 0.00001). Moderate heterogeneity was observed (I^2^ = 46%), suggesting low to moderate variability.

### 3.6 Subgroup analysis by sample source

**Figure 5:**
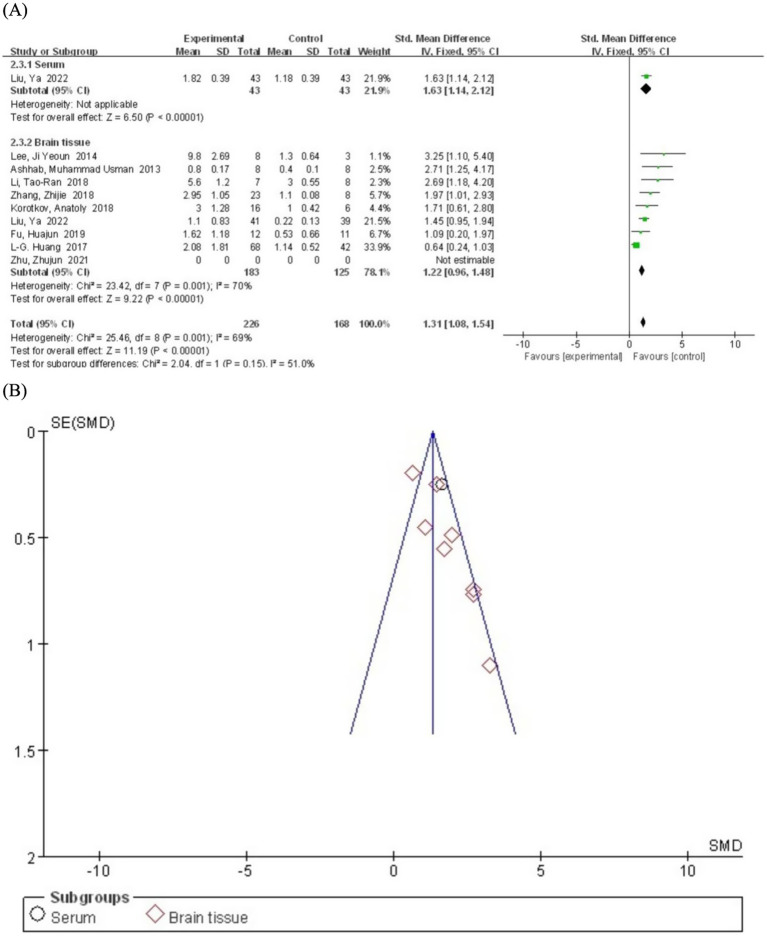
**(A)** Subgroup analysis by sample source category demonstrating the association between serum miR-155 level and epilepsy. **(B)** A funnel plot to assess publication bias.

We conducted subgroup analyses based on sample sources (serum and brain tissue) to explore potential sources of heterogeneity and differences in serum miR-155 levels between epilepsy patients and the control population. The serum subgroup included only one study. The results showed that miR-155 levels were significantly higher in epilepsy patients compared to the control group (SMD = 1.63; 95% CI, 1.14–2.12; *p* < 0.00001) (Figure 5). Heterogeneity assessment was not performed for the serum subgroup as it included only one study. The brain tissue subgroup comprised eight studies. The pooled analysis revealed that miR-155 levels were significantly elevated in epilepsy patients compared to the control group (SMD = 1.22; 95% CI, 0.96–1.48). However, significant heterogeneity was observed (I^2^ = 70%, df = 7), possibly due to differences in tissue collection methods, epilepsy subtypes, or experimental protocols across studies. Notably, 8 of the 9 included studies utilized brain tissue samples. While these specimens provide direct pathological evidence, their invasive nature substantially limits clinical applicability. In contrast, cerebrospinal fluid (CSF) - by virtue of its direct contact with neural pathology - may represent a superior biological matrix compared to peripheral blood. Although insufficient data precluded CSF analysis in the current study, its anatomical proximity to epileptogenic foci warrants prioritized investigation in future research.

## 4 Discussion

The accurate diagnosis and classification of epilepsy, combined with precise therapeutic interventions, are the cornerstones of optimal clinical management. Identifying biomarkers for early diagnosis is therefore crucial. Growing evidence implicates miRNAs in epileptogenesis through mechanisms including neuronal circuit reorganization, apoptosis, glial proliferation, neuroinflammation, and oxidative stress (29, 30). Unlike other epilepsy-associated miRNAs that primarily function in single pathways [e.g., miR-146a in neuroinflammation (31) or miR-132 in synaptic plasticity (32)], miR-155 exhibits multimodal regulation of epileptogenesis through simultaneous modulation of neuroinflammation, blood–brain barrier integrity, and neuronal excitability control. Consistent with prior findings, our clinical data confirm significantly elevated miR-155 level in epilepsy patients versus healthy controls. Ashhab et al. (17) reported coordinated upregulation of miR-155 and TNF-*α* across developmental stages in a rat model of status epilepticus and suggested their dual role in neuroinflammation and barrier dysfunction. Both TNF-α and miR-155 showed significant upregulation during all three stages of mesial temporal lobe epilepsy (MTLE). This co-expression pattern correlated with inflammatory responses and blood–brain barrier dysfunction—processes also implicated in autoimmune disorders, where miR-155 regulates immune activation. Lind et al. (33) reported that overexpression of miR-155 in dendritic cells can reduce SH2-containing inositol phosphatase (SHIP), thereby inducing an autoimmune response. Given the close association between autoimmunity and neurological disorders, particularly degenerative diseases, there appears to be a significant relationship between miR-155 and various neurological conditions.

**Figure 6:**
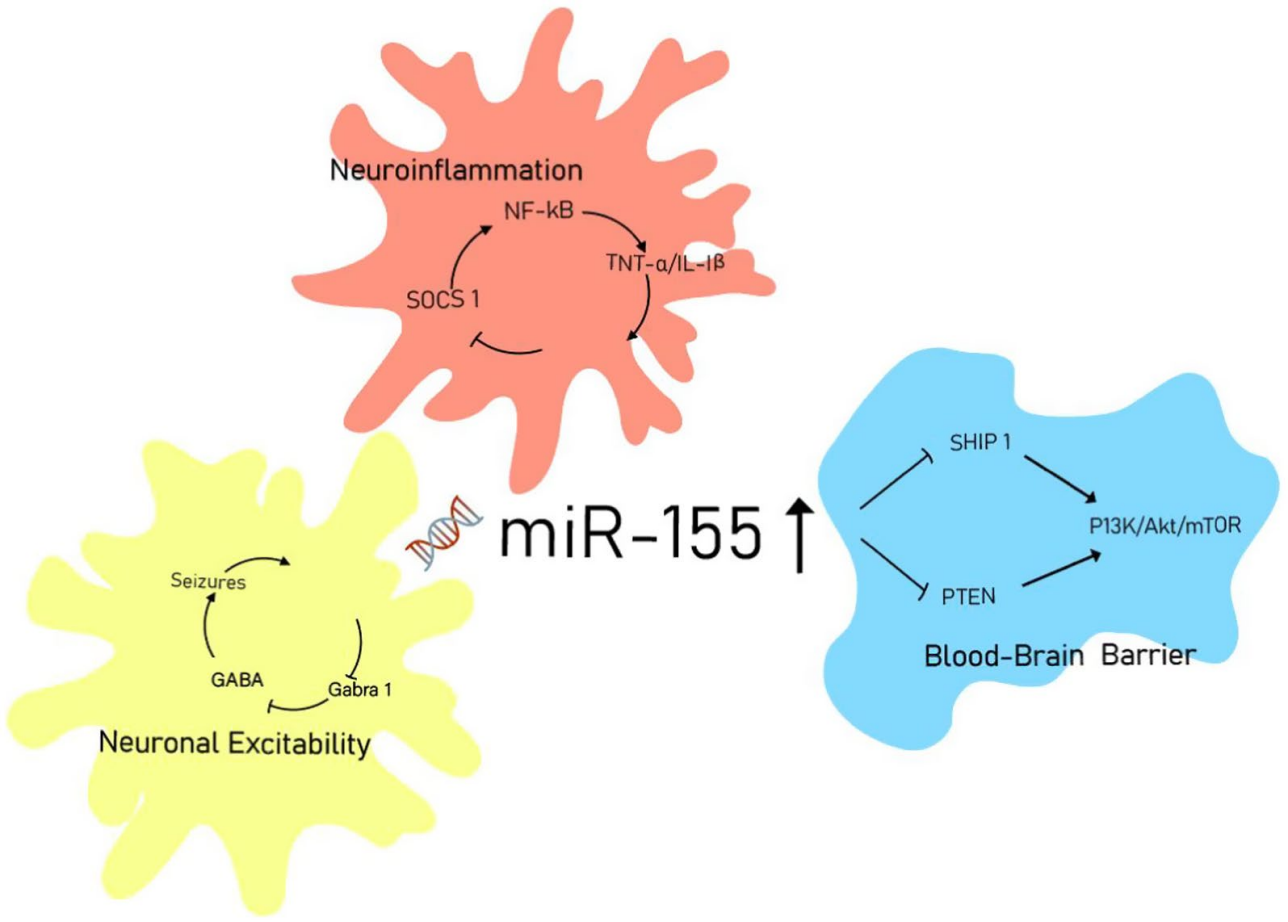
miR-155 signaling network in epileptogenesis.

Moreover, some studies have explored the signaling pathways of miR-155 in order to provide a more comprehensive understanding of its role in the pathogenesis of epilepsy. The phosphatidylinositol 3-kinase (PI3K)/protein kinase B (Akt) signaling pathway is an essential conduit for “survival signals” and is also referred to as the “antiapoptotic” pathway (34). Duan et al. (34) demonstrated that overexpression of miR-155 inhibits the PI3K/Akt signaling pathway in HT22 cells under glutamate stimulation. This finding suggests that miR-155 may participate in the pathogenesis of epilepsy through the PI3K/Akt/mTOR signaling pathway. However, this experimental approach differs from the methodologies described previously. Zhang et al. (15) demonstrated that the delivery of miR-155 inhibitors directly to the brain improved post-ischemic non-convulsive seizures (NCS) by modulating neuroinflammation and GABA signaling pathways in the parietal cortex, hippocampus, and amygdala. Clinical and animal studies by Huang et al. (19) indicated that silencing miR-155 may significantly suppress the pathological features associated with epilepsy by promoting the expression of Sesn3. Furthermore, animal experiments conducted by Zhou et al. (16) revealed that miR-155 plays a critical role in biological processes by negatively regulating multiple target mRNAs. These findings may offer new therapeutic targets for the treatment of epilepsy (Figure 6).

Notably, miR-155 may promote the development and progression of epilepsy by regulating RNA transcription. Research by Gong et al. (35) has demonstrated that miR-155 can modulate gene expression by regulating circular RNAs (circRNAs) during transcription or post-transcriptionally. CircRNA acts as a sponge that helps miR-155 enhance the expression of FOXO3a (forkhead box O3), thereby exerting its effects in temporal lobe epilepsy (TLE). Various miRNAs have shown dysregulation in brain tissue and serum samples from epilepsy patients, as well as in different animal models of this neurological disorder (36). Additionally, the role of genetic variations within miRNA-encoding genes in the risk of epilepsy or drug resistance to antiepileptic medications represents a promising area for further research exploration.

To the best of our knowledge, this study is the first systematic review and meta-analysis of the association between miR-155 and epilepsy. Current research indicates a positive correlation between elevated level of miR-155 and the occurrence of seizures, which is consistent with previous findings (32). This article reviews the existing literature on the association between miR-155 level in brain tissue and serum with epilepsy, and evaluates the potential of miR-155 as a biomarker for epilepsy. Among the 9 included studies, one lacked quantifiable data, resulting in a final analysis of 226 patients and 168 matched controls. The results indicate that economic status and social development appear to significantly influence the occurrence and progression of epilepsy. Improving economic development and social conditions can notably reduce the incidence of epilepsy and enhance the quality of life for patients (37, 38). However, the age subgroup analysis did not reveal clear evidence suggesting a direct relationship between the expression levels of miR-155 in epilepsy patients and age. Although age did not directly correlate with miR-155 expression, current evidence suggests that seizure-induced neuronal damage may be age-dependent, potentially indirectly influencing miR-155 levels.

Beyond demographic factors, clinical variables also significantly influence miR-155 expression, including seizure duration/frequency and antiepileptic drug regimens. Huang et al. (39) demonstrated that sodium valproate pretreatment reduced miR-155 upregulation during epileptic seizures, whereas immunomodulator use was associated with elevated miR-155 levels in post-traumatic epilepsy patients (40). However, the lack of standardized reporting of these variables in the included studies precluded subgroup analysis of these potentially important confounders.

Although the results of this meta-analysis suggest that miR-155 holds promise as a diagnostic biomarker for epilepsy, there are several limitations to consider. First, significant heterogeneity (I^2^ = 69%) was observed among included studies, potentially stemming from variations in patient demographics, epilepsy subtypes, and methodological differences in sample collection and detection protocols. Although subgroup analyses showed no significant effects (*p =* 0.31) of country, age, or sample source, these methodological variations may still introduce potential bias to the conclusions. Second, the geographical distribution of studies was imbalanced, with Chinese studies comprising 7 of the 9 included reports. While no significant inter-country differences were detected, this regional predominance may limit the generalizability of the findings. Third, the sample type distribution was highly skewed, with only one study using serum samples versus eight utilizing brain tissue, substantially constraining the evaluation of miR-155 as a non-invasive biomarker. Notably, CSF - which offers more direct pathological relevance due to its contact with neural tissues - could not be analyzed due to insufficient data, representing an important target for future investigation. Furthermore, inconsistent reporting of key clinical variables such as epilepsy classification, seizure frequency, and medication regimens, along with the lack of longitudinal data tracking miR-155 changes throughout disease progression or treatment response, significantly hinders a comprehensive assessment of its clinical significance. Collectively, these limitations define the prerequisite multicenter efforts needed to bridge miR-155 biomarker potential with clinical translation.

Building on these findings, the translational pathway for miR-155—from biomarker discovery to clinical implementation—requires addressing three interdependent challenges that emerged from our analysis: Although miR-155 shows significant potential as a biomarker, its clinical application faces multiple interrelated challenges in terms of technology, validation, and treatment: the instability of miR-155 in serum without RNase inhibitors (41), and the invasive nature of cerebrospinal fluid collection. These constitute significant technical obstacles. However, emerging exosome miRNA analysis methods may provide solutions (42). These technical limitations exacerbate the significant heterogeneity in miR-155 levels reported in our meta-analysis, and guiding principles for establishing epilepsy biomarker studies may provide a direction for solutions (43). Critically, preclinical studies using antagomirs have confirmed their therapeutic potential. Preclinical studies have demonstrated that intranasal delivery of miR-155-5p antagomir can reduce seizure severity and mitigate hippocampal damage (16). However, it must be emphasized that these findings are derived exclusively from animal models. Significant translational challenges remain, including blood–brain barrier permeability limitations and interspecies variations. Current discussions regarding targeted therapies such as antagomirs and intranasal delivery systems (44) should therefore be considered exploratory hypotheses requiring validation in human clinical trials.

## 5 Conclusion

In summary, miR-155 demonstrates potential as a diagnostic biomarker for epilepsy, facilitating early detection and elucidating disease pathophysiology. Furthermore, miR-155 could help classify epilepsy subtypes mediated by neuroinflammation. Notably, it represents a rare theranostic target whose elevation provides an actionable pathway for antagomir-based therapy. Current evidence is largely derived from animal studies, whose translational limitations—including interspecies biological differences and simplified disease models—must be acknowledged. Thus, although preclinical data establish mechanistic foundations, multi-center clinical trials with standardized protocols are imperative to validate the diagnostic utility of miR-155. Translating miR-155 research into clinical practice requires rigorous validation of its reliability as both a diagnostic biomarker and therapeutic target in human epilepsy, pending advances in CNS delivery technologies.

## Data Availability

The original contributions presented in the study are included in the article/supplementary material, further inquiries can be directed to the corresponding author.
